# Profiling Antibody Response Patterns in COVID-19: Spike S1-Reactive IgA Signature in the Evolution of SARS-CoV-2 Infection

**DOI:** 10.3389/fimmu.2021.772239

**Published:** 2021-11-03

**Authors:** Gabriel Siracusano, Chiara Brombin, Claudia Pastori, Federica Cugnata, Maddalena Noviello, Elena Tassi, Denise Princi, Diego Cantoni, Mauro S. Malnati, Norma Maugeri, Carla Bozzi, Gianni Saretto, Nicola Clementi, Nicasio Mancini, Caterina Uberti-Foppa, Nigel Temperton, Chiara Bonini, Clelia Di Serio, Lucia Lopalco

**Affiliations:** ^1^ Division of Immunology, Transplantation and Infectious Disease, Immunobiology of HIV Group, San Raffaele Scientific Institute, Milan, Italy; ^2^ University Centre of Statistics in the Biomedical Sciences, Vita-Salute San Raffaele University, Milan, Italy; ^3^ Experimental Hematology Unit, San Raffaele Scientific Institute, Milan, Italy; ^4^ Viral Pseudotype Unit, Medway School of Pharmacy, Universities of Kent and Greenwich, Chatham, United Kingdom; ^5^ Viral Evolution and Transmission Unit, San Raffaele Scientific Institute, Milan, Italy; ^6^ Autoimmunity and Vascular Inflammation Unit, San Raffaele Scientific Institute, Vita-Salute San Raffaele University, Milan, Italy; ^7^ Fondazione Opera San Camillo, Milano, Italy; ^8^ Laboratory of Medical Microbiology and Virology, Vita-Salute San Raffaele University, Milan, Italy; ^9^ Infectious Diseases Clinic, Vita-Salute San Raffaele University, Milan, Italy; ^10^ Experimental Hematology Unit, San Raffaele Scientific Institute, Vita-Salute San Raffaele University, Milan, Italy; ^11^ Biomedical Faculty, Università della Svizzera Italiana, Lugano, Switzerland

**Keywords:** COVID-19, neutralizing antibodies, clinical outcome, VOC, SARS-CoV-2

## Abstract

This contribution explores in a new statistical perspective the antibody responses to severe acute respiratory syndrome coronavirus 2 (SARS-CoV-2) in 141 coronavirus disease 2019 (COVID-19) patients exhibiting a broad range of clinical manifestations. This cohort accurately reflects the characteristics of the first wave of the SARS-CoV-2 pandemic in Italy. We determined the IgM, IgA, and IgG levels towards SARS-CoV-2 S1, S2, and NP antigens, evaluating their neutralizing activity and relationship with clinical signatures. Moreover, we longitudinally followed 72 patients up to 9 months postsymptoms onset to study the persistence of the levels of antibodies. Our results showed that the majority of COVID-19 patients developed an early virus-specific antibody response. The magnitude and the neutralizing properties of the response were heterogeneous regardless of the severity of the disease. Antibody levels dropped over time, even though spike reactive IgG and IgA were still detectable up to 9 months. Early baseline antibody levels were key drivers of the subsequent antibody production and the long-lasting protection against SARS-CoV-2. Importantly, we identified anti-S1 IgA as a good surrogate marker to predict the clinical course of COVID-19. Characterizing the antibody response after SARS-CoV-2 infection is relevant for the early clinical management of patients as soon as they are diagnosed and for implementing the current vaccination strategies.

## Introduction

In December 2019, the severe acute respiratory syndrome coronavirus 2 (SARS-CoV-2) was identified as the causative agent of the coronavirus disease 2019 (COVID-19). Nowadays, the pandemic remains a dramatic global challenge due to the unpredictable disease outcome and the rapid emergence of genetic variants. It became clear very quickly that COVID-19 was characterized by highly variable clinical manifestations ranging from asymptomatic to mild and moderate, while progressing to respiratory and multiorgan failure in certain patients, even leading to death (1).

IgM, IgA, and IgG targeting the viral spike (S) and nucleoprotein (NP) are sequentially or concomitantly generated promptly after infection (2, 3). Antibody production reduces the risk of severe disease and neutralizing antibodies (nAbs) represent important correlates of protection against viral infections (4). Several studies examined the magnitude, the dynamic, the persistence, and the functions of SARS-CoV-2-specific antibodies; however, the evidences are not concordant across the studies (5–7).

The antiviral response towards SARS-CoV and MERS-CoV remained detectable for an average of 2 years (8) and less than 1 year (9) after primary infection, respectively. The persistence of SARS-CoV-2-protective antibodies is still under investigation.

Given the heterogeneity of COVID-19, identifying biomarkers, within a multivariate perspective, plays a crucial role in early diagnosis, monitoring, and management of patients, complementing the clinical assessment of the disease. Current available biomarkers (10) attempted at identifying subjects at high risk to develop the disease, and they allowed confirming the diagnosis, assessing the severity, discriminating the requirement of hospital care, driving the administration, and the response to therapy. However, their performance in terms of clinical utility remains to be evaluated and the discovery of novel biomarkers is a clinical unmet need.

In the recent literature, data-driven approaches have been proved as very sensitive in identifying risk factor effects for clinical outcomes in COVID-19 case series data collected with no design in emergency conditions (11, 12). We here apply machine-learning algorithms to profile the antibody landscape of a heterogeneous SARS-CoV-2-infected cohort of patients, thus identifying specific signatures allowing to drive the early diagnosis and predict the clinical course of COVID-19.

We also assessed the longevity of the naturally induced antibodies against the wild-type virus and their capability to cross-neutralize the current circulating variants of concerns.

## Materials and Methods

### Patient Selection and Data Collection

Plasma samples from 141 SARS-CoV-2-infected subjects between February and May 2020 admitted to the Emergency or Clinical departments of the Istituto di Ricovero e Cura a Carattere Scientifico (IRCCS) San Raffaele Hospital or healthcare workers from care-home residents were included in this study. The inclusion criterion for this clinical-biological case series study is provided in the **SI Appendix**.

### Cell Line

Human embryonic kidney 293 cells (HEK 293T/17 cells) were acquired from Programme EVA Centre for AIDS Reagents, National Institute for Biological Standards and Control (NIBSC, UK), cultured in Dulbecco’s modified Eagle’s medium (DMEM) supplemented with 4.5 mg/ml glucose, 2 mM L-glutamine (Lonza, Basel, Switzerland), 100 units/ml penicillin-streptomycin (Lonza) and 10% of FBS (Euroclone, Milan, Italy). The cells were incubated at 37°C, 5% CO_2_ in humidified atmosphere.

### Production and Titration of SARS-CoV-2 Lentiviral-Pseudotyped Particles

SARS-CoV-2-pseudotyped particles were produced and titrated as previously described (13) and briefly detailed in the **SI Appendix**.

Neutralization assays were performed by incubating 106 RLU of pseudotyped viruses with endpoint twofold serial dilutions of heat-inactivated plasma samples at 37°C 5% CO_2_ for 1 h before addition of 104 HEK 293T/17-ACE2/TMPRSS2 cells per well. After 72 h at 37°C, the cells were lysed in Luciferase Assay (Promega, Madison, WI, USA) and luciferase activity was measured using a Victor luminometer. Neutralization titers were expressed as IC_50_ values, defined as the concentration of plasma required to achieve half maximal neutralization.

### Purification of IgG and IgA

HiTrap Protein G HP 1 ml column (GE Healthcare, Boston, MA, USA) and Peptide M-Agarose 2 ml column (InvivoGen Europe, Toulouse, France) were used to purify IgG and IgA, respectively, from 300 µl of plasma according to the manufacturing instructions and using an automatic HPLC system (Biologic DuoFlow, Bio-Rad Laboratories, Hercules, CA, USA).

### Detection of SARS-CoV-2-Specific Antibodies

IgA, IgG, and IgM specific for the S1 and S2 subunits were detected in plasma samples as previously described in an in-house ELISA (13). Briefly, recombinant S1 or S2 protein (Abeomics, San Diego, CA, USA) was plated at 0.1 µg/well on Maxisorp 96-well plates (Thermo Scientific, Waltham, MA, USA) and incubated overnight at 4°C in 50 mM carbonate/bicarbonate buffer at pH 9.5. After blocking for 1 h at 37°C with PBS containing 10% BSA and 0.05% Tween 20, duplicate of 1:100 diluted plasma samples were plated and incubated for 1 h at 37°C. When optical density (OD) values were higher than 2.0, the samples were further diluted and retested. Plasma samples from 40 healthy donors collected before COVID-19 pandemic were used as negative controls. The plates were then treated for 30 min at 37°C with 1:6,000 horseradish peroxidase (HRP)-conjugated goat antihuman IgA, antihuman IgG, or antihuman IgM (Southern Biotech, Birmingham, AL, USA). Plates were developed using TMB 2C (KPL-SeraCare, Milford, MA, USA), and the reaction was stopped with 10% sulfuric acid after 5 min. The OD values were read at wavelengths of 450 and 620 nm using a PowerWave ELISA reader (BioTek, Winooski, VT, USA). The cutoff value was determined by calculating the mean OD + 3SD obtained with healthy individuals.

IgG, IgA, and IgM to the NP were measured through SARS-CoV-2 (COVID-19) ELISA Kits (Novatec, Baltimore, MD, USA) according to the manufacturer’s instruction.

An initial screening for NP reactivity was performed to evaluate positive candidate control samples for S1- and S2-binding assays. One high- and one mid-reactive samples were chosen as shown in **Supplementary Table S5**.

Cutoff values were arbitrarily established to correspond to 10 Arbitrary Units (AU) for both the in-house ELISA and Novatec ELISA kits, and the specificity for all of the assays ranged from 98.26% to 100%. The AU were calculated with the formula: [mean OD (sample) × 10 AU (cutoff)]/mean OD (cutoff).

The WHO International Standard (WHO IS, National Institute for Biological Standards and Control, NIBSC, UK, cod. 20/136) and the WHO Reference Panel (WHO RP, NIBSC, cod. 20/268) for anti-SARS-CoV-2 antibody were tested at 1:100 dilution in the in-house ELISA for S1 and S2 and the Novatec kit for NP as described above, to check the concordance with our results (see **Supplementary Table S5A**). The AU values obtained were then transformed in International Units per milliliter (IU/ml) relative to WHO IS 20/136 using the formula [AU (sample) × 1,000 (IU/ml)]/AU (WHO IS) (see **Supplementary Table S5B**), and the conversion factors calculated were the following: 58.2 for anti-S2 IgG, 76.5 for anti-S2 IgA, 47.5 for anti-S1 IgG, 80.8 for anti-S1 IgA, 19.9 for anti-NP IgG, and 39.4 for anti-NP IgA. The AU for IgM were not transformed in IU/ml since WHO IS showed no reactivity in the S1-, S2-, NP-IgM-binding assays.

### Statistical Analysis

Median, interquartile range, minimum, and maximum values have been used to summarize quantitative variables; frequency distributions have been reported for categorical variables. Spearman’s correlation coefficient has been used to examine the relationship among quantitative variables.

Friedman test followed by Dunn’s multiple comparison tests has been applied in the presence of repeated measures.

Tobit regression models have been estimated to model left censored dependent variable.

Classification and Regression Trees (CART) analyses have been performed to identify those variables that best discriminate patients with different outcomes.

Linear mixed-effects models have been applied to examine the dynamic of IC_50_ over time. A random effect was specified on patients’ ID, thus leading to random intercept models. Backward model selection procedures have been applied to obtain a smaller set of relevant covariates, and *post-hoc* analyses have been implemented to compare responses of different groups of patients at a fixed time point. Detailed statistical methods are reported in the **SI Appendix**.

## Results

### Patient’s Cohort

The cohort consisted of 141 COVID-19 individuals enrolled between February and August 2020, including patients admitted at San Raffaele Hospital in the COVID-19 clinical-biological case series study (COVID-BioB) (ClinicalTrialsgov identifier NCT04318366), and healthcare workers from care-home residents in Lombardia, Piemonte, and Liguria regions (**Table 1**). The median age was 56 years (range 24–94), and males and females were balanced distributed (48.2% and 51.8%, respectively). They experienced a wide range of clinical manifestations, ranging from asymptomatic (13.5%), mild (36.9%, WHO scores 1 and 2), moderate (34%, WHO scores 3 and 4), and severe (15.6%, WHO scores 5 and 6) symptoms. Among them, 51.1% required hospital care and 11.3% passed away. Plasma samples were collected at a median of 38.40 days postsymptoms onset (PSO, range 1–252 days). When grouped by time PSO, 54 subjects contributed to an early plasma sample collected within 1 month (23 subjects at 0–7 days and 31 subjects at 8–30 days), 21 subjects contributed a 31–60-day sample, 28 contributed a 2–4-month sample, and 19 contributed a 5–9-month sample. Furthermore, 72 subjects provided longitudinal plasma samples, of which 51 contributed a 2 time points and 21 contributed 3 time points.

**Table 1:** Demographic and clinic characteristics of the cohort.

Total	141
**Demographics**	
Age (median [IQR], range)	56.00 [47.00, 67.00] (24–94)
Sex (% male)	48.2 (68/141)
**Clinical characteristics**	
Asymptomatic (%)	13.5 (19/141)
WHO score	
1 (%)	17 (24/141)
2 (%)	19.9 (28/141)
3 (%)	12.1 (17/141)
4 (%)	22 (31/141)
5 (%)	6.4 (9/141)
6 (%)	9.2 (13/141)
Days PSO to sample collection (median [IQR], range)	38.40 [9.25, 107.62] (1–252) [*n* = 122]
Hospitalized (%)	51.1 (72/141)
Days hospitalization (median [IQR], range)	18.5 [9.75, 35.75]
ICU (%)	10.6 (15/141)
Deceased (%)	11.3 (16/141)

### SARS-CoV-2-Specific Antibody Responses Span From Undetectable to Robust

**Figure 1:**
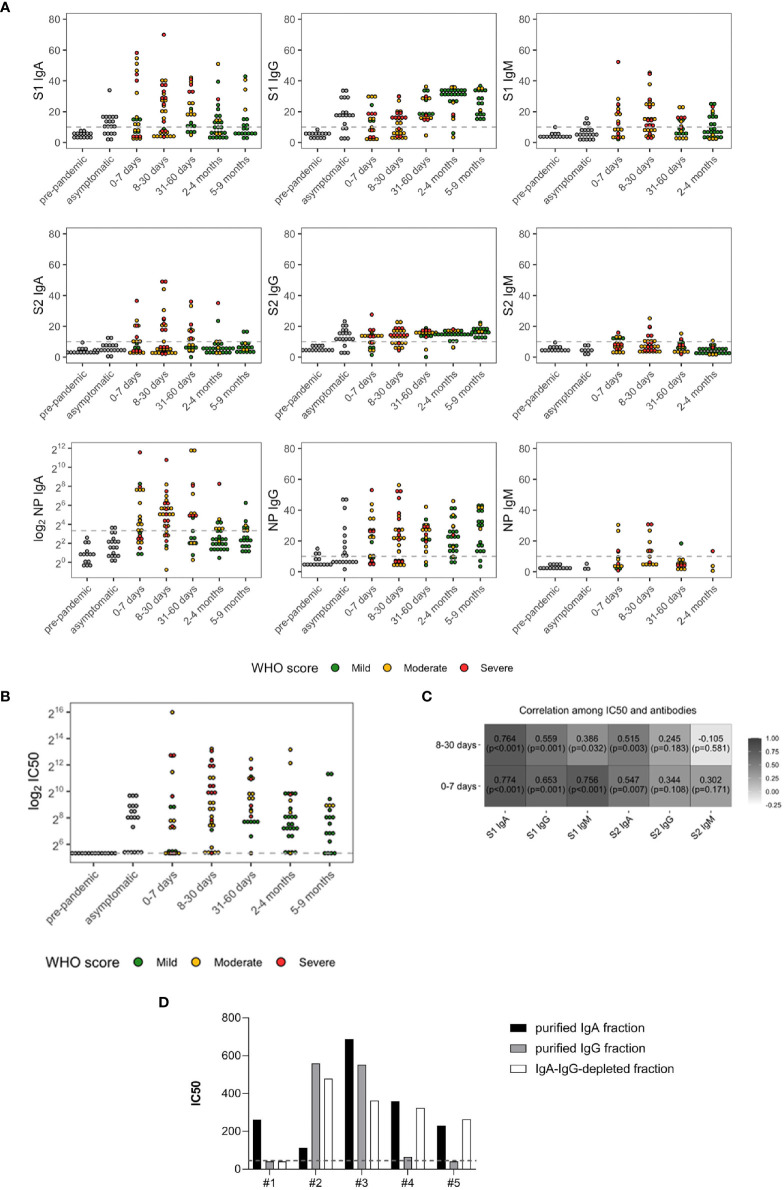
SARS-CoV-2 antibody levels **(A)** and neutralizing antibody titers (IC_50_) **(B)** in prepandemic (*n* = 14), asymptomatic (*n* = 19), and symptomatic subjects (*n* = 122). The latter were grouped based on days PSO and color coded according to the severity of the disease: green (mild), yellow (moderate), and red (severe). **(C)** Spearman’s correlations between IC_50_ and SARS-CoV-2 antibodies in the symptomatic subjects with onset of symptoms of less or equal to 7 days (*n* = 23) and between 8 and 30 days (*n* = 31). **(D)** IC_50_ of IgA- and IgG-purified fractions and IgA/IgG-depleted fractions from five randomly selected patients. The dashed lines represent the cutoff of the ELISA tests (10 AU) or the lowest dilution (1:40) of the neutralization assay.

We first investigated the development of binding IgA, IgM, and IgG towards the NP and the S subunits, S1 and S2. The limit of detection of the tests was set equal to 10 based on values detected for 120 healthy individuals sampled in 2018, and 14 were randomly chosen as negative controls (referred as prepandemic). The magnitude of the individual antibody response was heterogeneous among the cohort stratified according to the time of sampling PSO (**Figure 1A**). A fraction of both asymptomatic and symptomatic patients (1.4%) did not develop any antibody response. The severity of the disease did not affect antibody development, as suggested by comparable levels between asymptomatic infections and subjects with mild or more severe symptoms. Asymptomatic individuals developed lower detectable levels of anti-NP antibodies compared with symptomatic within 9 months PSO; 73.7% of asymptomatic subjects elicited S1 IgG (median 18.11) and 78.9% developed S2 IgG (median 12.51). The magnitude of the S1- and S2-specific IgG response was comparable with that found in symptomatic subjects. S1-, S2-, and NP-specific IgM in asymptomatic individuals were undetectable at the time of sampling from the positive swab (range 40–143 days from the positive swab). The proportion of asymptomatic individuals with detectable levels of anti-S1 IgA (63.2%) was higher than those reporting symptoms and collected between 0 and 7 days PSO (56.5%), and their levels were comparable (median 11.48 and 12.68, respectively).

All the three Ig isotypes were developed within 0–7 days PSO, as previously reported (4, 14). Anti-S1 IgA, IgG, and IgM were detected in 56.5% (median 12.68), 47.8% (median equal to 10), and 52.2% (median 12.17) of patients, respectively. Anti-S2 IgA, IgG, and IgM were developed in 34.8%, (median equal to 10), 60.9% (median 12.57), and 31.8% (median equal to 10) of patients, respectively. IgA and IgG towards NP were the most representative isotypes developed at this time point (**Supplementary Table S1**), detected in 56.5% (median 14.78) and 60.9% (median 21.98) of patients, respectively, whereas only 30.8% developed NP-IgM (median equal to 10).

Between 8 and 30 days PSO, 74.2% of patients developed higher anti-NP IgA levels than those sampled at 0–7 days PSO (median 32.63). NP-IgM were higher compared with the previous time point as well (median 11.63) in the 50% patients. Differently from antibodies specific for NP, the percentage of patients developing anti-S1 isotypes was comparable with that analyzed within 7 days PSO, even their levels were higher [54.8% for IgA (median 15.67) and IgG (median 12.89), 64.5% for IgM (median 12.34)]. Similar proportions were observed for S2-IgA [38.7% (median equal to 10)] and S2-IgG [67.7% (median 13.68)], whereas 23.3% developed IgM (median equal to 10). Of note, patients enrolled during this time point mainly experienced moderate (61.3%) and severe (35.5%) symptoms.

Between 31 and 60 days PSO, both the percentage of patients with IgA and IgG towards S1 and S2 and their respective levels increased (71.4% (median 19.28) and 95.2% (median 18.38) for S1 and 47.6% (median 9.07) and 90.5% (median 15.62) for S2). Conversely, only 42.9% and 16.7% of patients had detectable levels of S1- (median equal to 10) and S2-specific (median equal to 10) IgM, respectively. Compared with the early week PSO, only 52.4% of patients, mainly with mild symptoms, developed anti-NP IgA (median 25.63).

Between 2 and 4 months, sustained levels of S1-, S2-, and NP-specific IgG were detected in the majority of patients (92.9% (median 31.49), 89.3% (median 15.32), and 85.7% (median 23.51), respectively). Patients sampled between 5 and 9 months PSO had similar antibody levels, with a slight decrease in anti-S1 IgG (100% (median 26.65) for S1, 100% (median 16.64) for S2, and 89.5% (median 29.55) for NP). Conversely, specific IgA levels decreased (median equal to 10 for S1, S2, and for NP).

Of the subjects, 80.9% developed antibodies with neutralizing activity (**Figure 1B**), and a broad variation in nAb titers was observed. Median plasma IC_50_ titer was 274.14 (97.00, 831.00), and 19.1% of patients did not reach the 50% neutralization at the lowest 1:40 dilution.

S1-reactive IgM and IgA significantly and strongly correlated with plasma neutralization (*r* = 0.756 and *r* = 0.774, respectively, both *p* < 0.001) within 7 days PSO, and the correlation was maintained for S1-IgA within 1 month PSO (*r* = 0.764, *p* < 0.001) (**Figure 1C**), suggesting a role for the early systemic IgA response in driving neutralization. To corroborate these results, we tested the neutralizing potential of IgG- and IgA-purified fractions from five randomly selected moderate and severe patients sampled within 30 days PSO. We confirmed the correlation between binding antibodies and neutralization for all IgA-purified fractions and for three out of five IgG-purified fractions. Of note, antibody-depleted fractions had neutralizing activity, probably due to soluble factors produced during the cytokine storm triggered in COVID-19 patients and IgM (**Figure 1D**).

We next wondered whether the baseline SARS-CoV-2-specific antibodies drive neutralizing activity. To address this question, Tobit regression models have been estimated on the 122 symptomatic COVID-19 subjects to investigate the role of undetectable or detectable antibody levels (values below the cutoff, ≤10; values above the cutoff, >10, respectively) on neutralization, while accounting for time PSO, age, sex, and hospitalization. We found that subjects who developed IgA, IgG, and IgM towards S1 and IgA and IgG towards S2 above the established cutoff of 10 had higher IC_50_ values compared with that with antibodies below the cutoff. Conversely, S2-reactive IgM did not affect the development of nAbs (*p* = 0.5382, **Supplementary Table S2**).

### Hospitalization, SARS-CoV-2 Antibody Response, and Neutralization Activity

**Figure 2:**
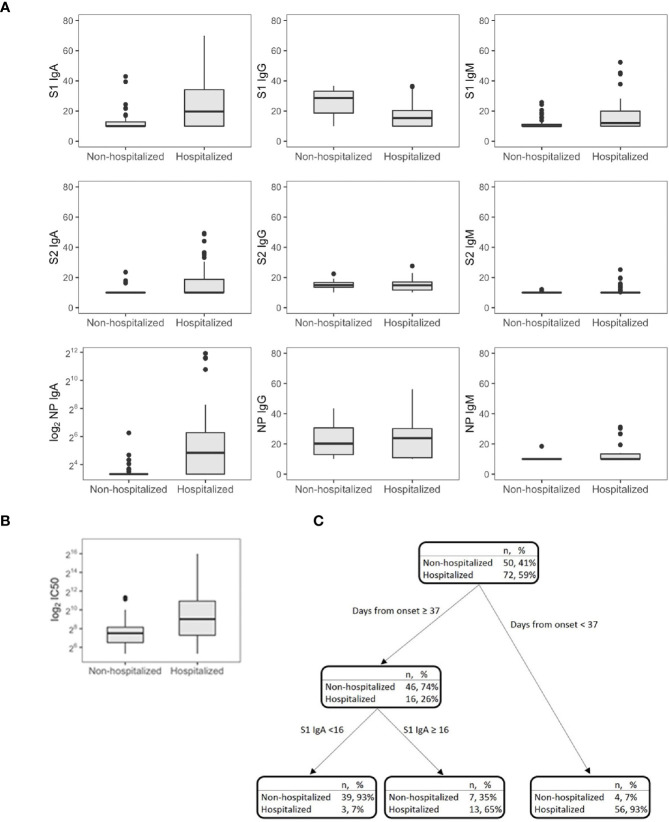
SARS-CoV-2 antibodies **(A)** and IC_50_ **(B)** in symptomatic nonhospitalized (*n* = 50) and hospitalized subjects (*n* = 72). Decision tree for the classification of the hospital admission in symptomatic subjects (*n* = 122) **(C)**.

Once more, Tobit regression models have been estimated to investigate the role of hospitalization in predicting SARS-CoV-2 antibody response while accounting for age, sex, and time PSO (**Supplementary Table S3**; **Figures 2A, B**). We found a significant and positive effect of hospitalization on anti S1-IgA (*p* = 0.0035) and NP-IgA (*p* = 0.0024), while hospitalization played a significant and negative effect on anti-S1-IgG (*p* = 0.0053). In addition, age significantly affected anti-S1 IgA (*p* = 0.007), IgM (*p* = 0.009), NP-IgG (*p* = 0.009), and NP-IgA (*p* = 0.009) levels and IC_50_ (*p* = 0.0028, transformed with base 2 logarithm). Time PSO had an impact on the development of IgG response towards S1 (*p* = 0.0036), S2 (*p* < 0.001), and NP (*p* = 0.0037) and specific IgM response towards S1 (*p* = 0.0385) and NP (*p* = 0.0163). Gender was a significant feature for the development of S1-reactive IgM only (*p* = 0.0297).

**Figure 3:**
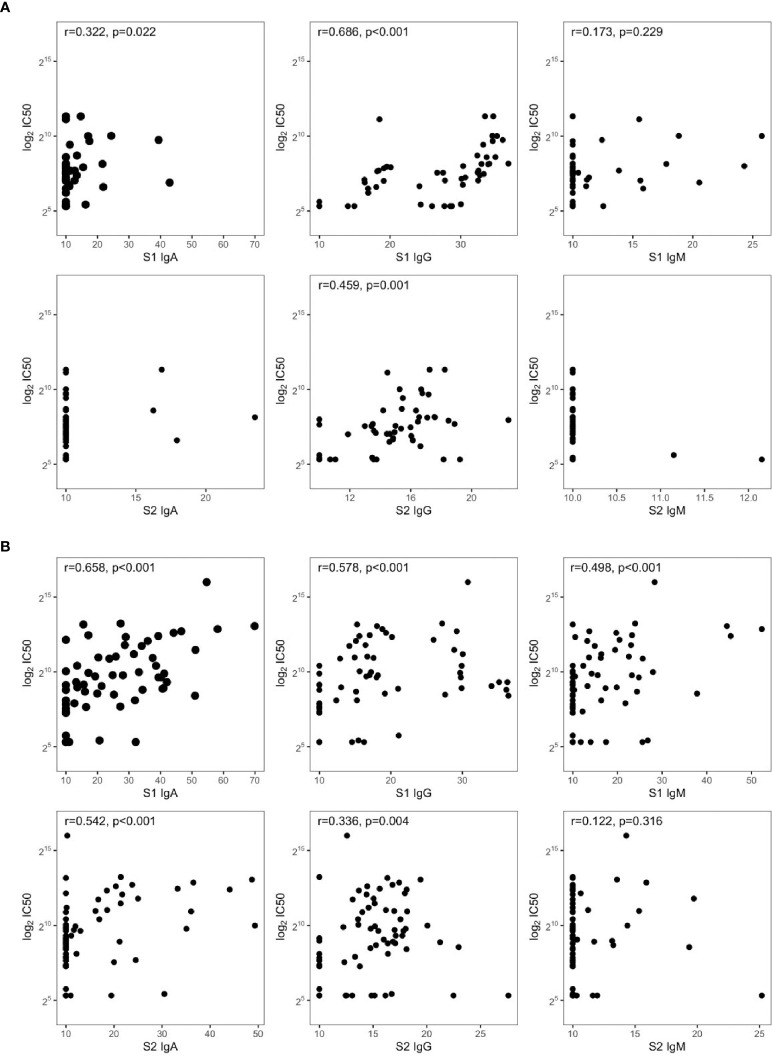
Spearman’s correlations between IC_50_ and SARS-CoV-2 antibodies in symptomatic **(A)** nonhospitalized (*n* = 50) and **(B)** hospitalized subjects (*n* = 72).

CART has been used to discriminate the risk of hospitalization. Among patients sampled after 37 days PSO, the risk was higher for those having S1 IgA levels equal or higher than 16 AU (65%, **Figure 2C**), while for those having S1 IgA levels lower than 16 AU, the risk was equal to 7%. Among nonhospitalized patients, anti-S1 IgG better correlated with neutralizing activity (*r* = 0.686, *p* = 0.001) than anti-S2 IgG (*r* = 0.459, *p* = 0.001) and anti-S1 IgA (*r* = 0.322, *p* = 0.022, **Figure 3A**). In these subjects, the magnitude of the antibody response did not have any impact on the resolution of the symptoms (**Supplementary Figure S1**).

S1-reactive antibodies correlated with neutralization in hospitalized patients, with S1-IgA playing the dominant role as nAbs (*r* = 0.658, *p* < 0.001, **Figure 3B**). Of note, this is in line on what was observed in **Figure 1**, since hospitalized patients were collected within 30 days PSO, when the S1-specific IgA response was higher and predictive for neutralization.

### S1-Specific IgA Levels Drive the Clinical Course of COVID-19

**Figure 4:**
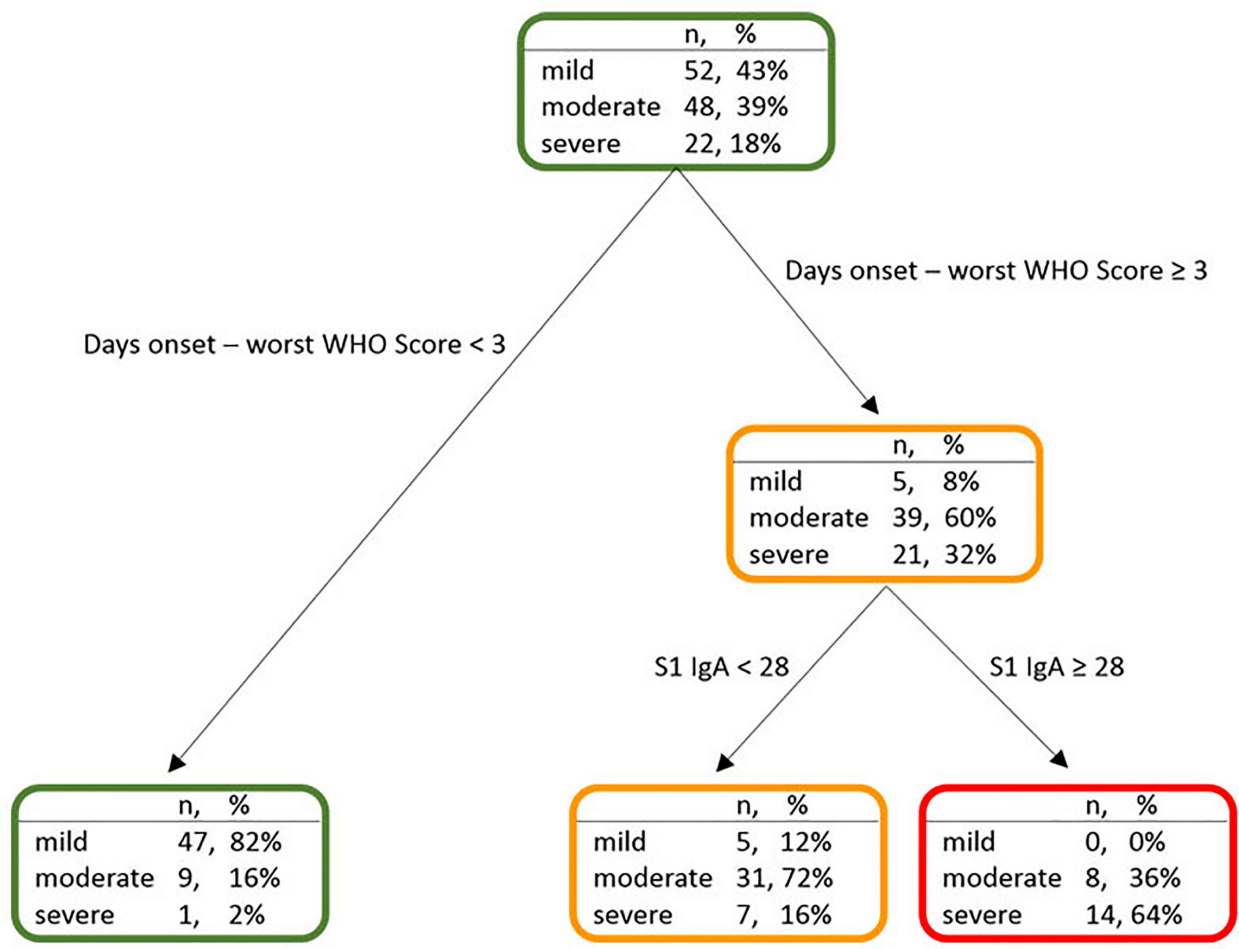
Decision tree for the classification of the WHO score in symptomatic subjects (*n* = 122).

**Figure 5:**
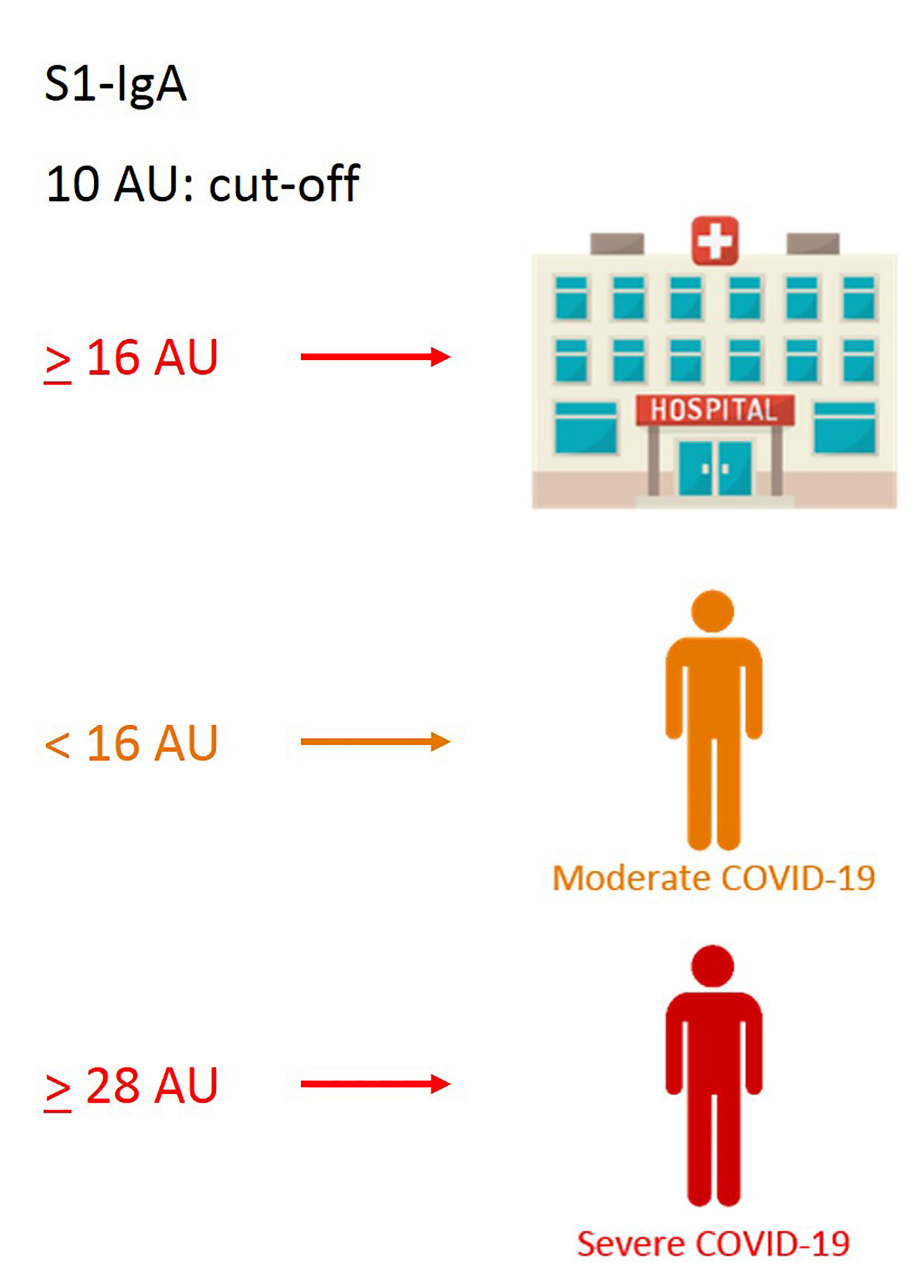
Graphical sketch of CART analyses showing relevant cutoff values of S1-IgA, allowing discrimination of the broad variety of COVID-19 clinical manifestations.

We next evaluated whether clinical and immunological characteristics of patients allow characterizing the COVID-19 course. An additional decision tree was derived to classify patients with different COVID-19 symptoms (**Figure 4**). Among all the input variables, the algorithm selected both time from disease onset to worst score and S1-IgA as those variables best discriminating among patients’ groups. Indeed, severe patients had a time from symptoms onset to worst score longer than or equal to 3 days and IgA towards S1 equal to or larger than 28 AU. Moderate patients were characterized by time from symptoms onset to worst score above 3 days but IgA towards S1 lower than 28 AU. Following the tree branches, S1-IgA lower than 28 AU allowed classifying 72% of patients with moderate symptoms. Conversely, S1-IgA higher than 28 AU allowed classifying 64% of subjects with severe disease. This parameter was not pivotal for classifying patients with mild disease. As a matter of fact, most of them were classified using the rule of a “time from disease onset to worst score lower than 3 days” (**Figure 4**). Therefore, S1-IgA are a good surrogate marker to discriminate the broad variety of clinical manifestations of COVID-19 early after the symptoms onset (**Figure 5**).

### Persistence of the Antibody Response to SARS-CoV-2 Up to 9 Months

**Figure 6:**
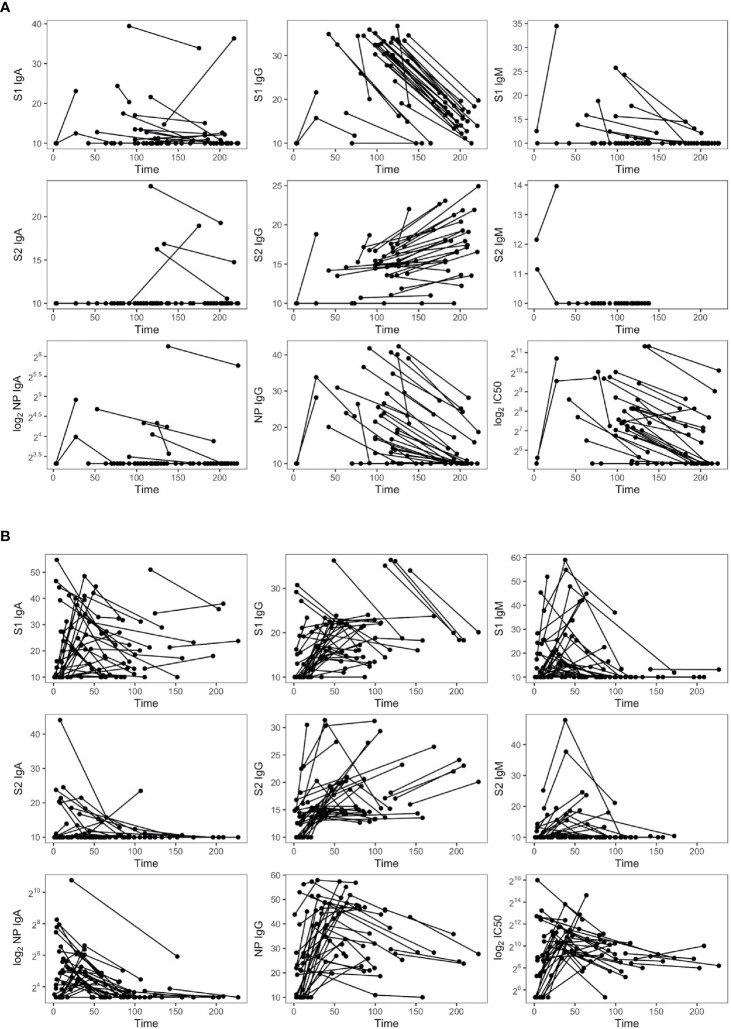
Dynamic of SARS-CoV-2 antibodies in nonhospitalized (*n* = 35) **(A)** and hospitalized subjects (*n* = 37) **(B)**.

We explored the duration of the antibody response against SARS-CoV-2 in 72 longitudinally symptomatic recovered patients requiring or not hospitalization (**Figure 6**). As expected, anti-S1 and S2-IgM declined up to undetectable levels in the majority of the two groups, even though three out of 24 nonhospitalized subjects maintained sustainable levels of S1-IgM up to 6 months PSO. A similar tendency was observed for anti-S1 IgA, except for some hospitalized patients who enhanced S1-IgA production even after 8 months PSO. Both hospitalized and nonhospitalized individuals lost S2-IgA, except for four nonhospitalized subjects who maintained high levels even after 7 months. Anti-NP IgA and IgG dramatically decreased in both groups but remained detectable even after 7 months PSO. Overall, SARS-CoV-2 IgM and IgG decreased during the follow-up period in infected subjects, especially those with high antibody levels at the first time point. Strikingly, while S1-IgG dramatically declined, S2-IgG enhanced over the same period of time (100–226.8 days) in both patient’s groups, pointing out the importance of the immunoreactivity towards S2.

**Figure 7:**
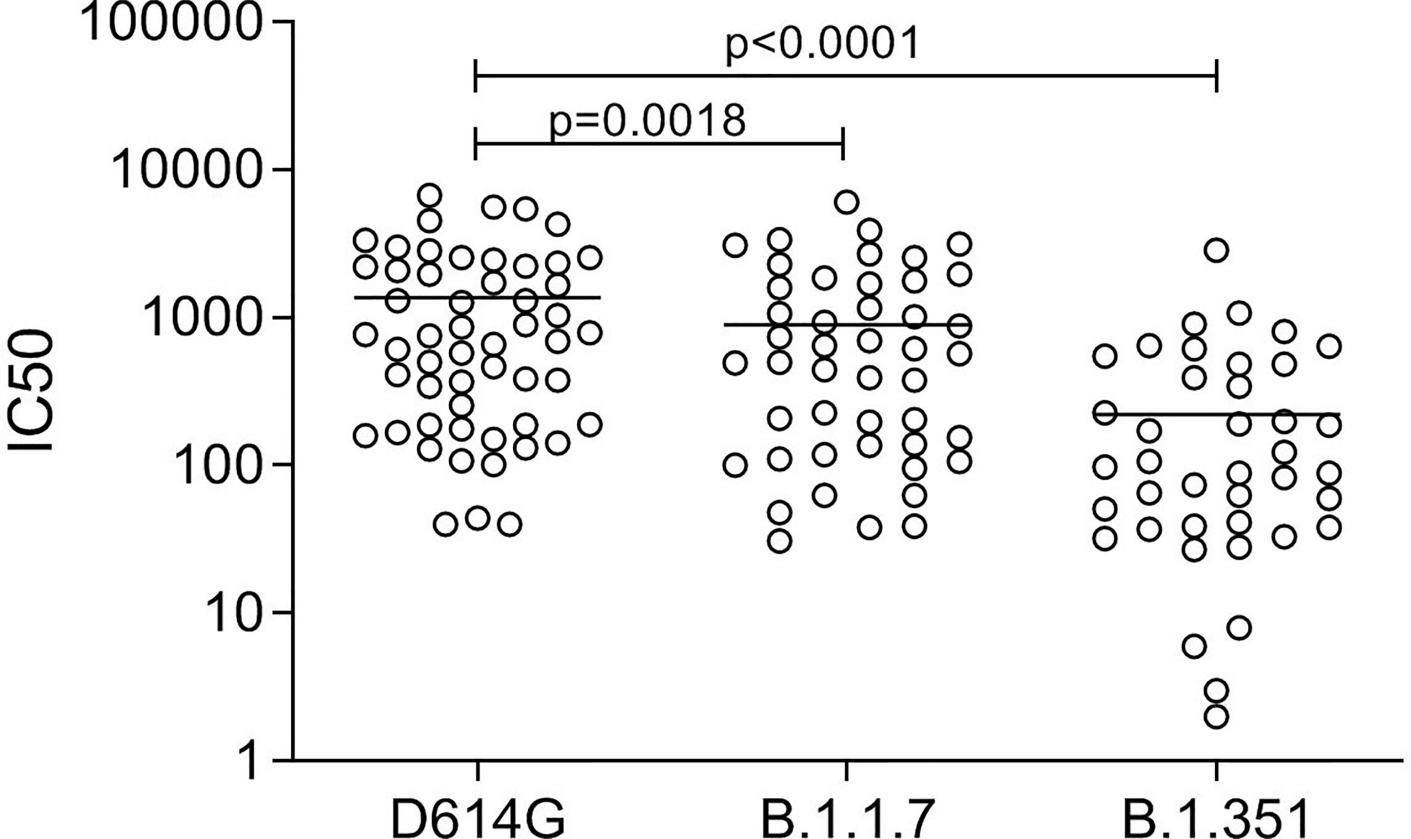
Neutralizing antibody titers in convalescent patients (*n* = 54) against the D614G spike and the spike protein of the variant B.1.1.7 and B.1.351. Friedman test followed by Dunn’s multiple comparison test was applied.

NAb titers declined but were still detectable in 11 out of 24 patients not requiring hospitalization and having a sampling time PSO higher than 6 months. Therefore, nAbs mirrored the kinetic of the antibody response, with the exception of S2-IgG. A panel of plasma samples with a range of neutralization titres (40–9,850 IC_50_) was tested for their ability to neutralize the B.1.1.7 and B.1.351 genetic lineages. Antibodies raised against the virus circulating during the first wave of the pandemic reduced their neutralizing activity with fold change of 1.7 and 7.2, respectively (**Figure 7**), as previously reported (15).

Based on considerations on sample size, we then focused on patients with moderate symptoms to investigate whether the baseline antibody response (positive/negative) might drive the nAb response in the longitudinal analysis. Twenty-three patients with at least two measurements at two different time points were considered. Time points were categorized by taking into account three conditions: T0 if the time of sampling from symptom onset was lower than 30 days, T1 if the time comprised between 30 and 60 days, and T2 if the time was larger than 60 days. Linear mixed effects models (LME) were estimated to model nAb activity over time. Along with group and time indicator variables, included as main effects as well in interaction, gender and age were entered in the model. The LME model revealed that the baseline levels of S1 and S2-IgA, S1-IgG, and S1-IgM played a role in shaping nAb activity dynamics.

*Post-hoc* analyses were performed to compare IC_50_ of patients with different baseline levels of antibodies at a fixed time point. It emerged as a significant difference at the first time point only (*p* = 0.001 for S1-IgA, *p* = 0.0034 for S2-IgA, *p* = 0.0017 for S1-IgG, and *p* = 0.0185 for S1-IgM, **Supplementary Table S4**).

## Discussion

We proposed a new methodological approach for identifying homogeneous profiles of patients’ antibody responses within a precision-medicine perspective thus accounting for individual sources of variability, thus providing the antibody landscape of patients mirroring the wide spectrum of clinical manifestations of SARS-CoV-2 infections.

Nearly all patients developed all classes of antibodies within the 7 days after the symptoms appeared. Consistent with our and other previous studies (2, 3), a fraction of both asymptomatic and symptomatic patients were seronegative. In accordance with previous studies (5–7), both the magnitude of the individual antibody response and neutralizing activity displayed great variability across the cohort, regardless of the severity of the disease. Reynolds et al. did not find any difference in the potency of nAbs from asymptomatic and mild patients, whereas lower T-cell response was a distinct feature of asymptomatic subjects (16). Severe COVID-19 cases associated with higher antibody production and neutralization titers (17, 18). We confirmed this evidence for all the antibody classes with the exception of S1 and S2 specific IgG levels. Indeed, in contrast with Long et al. (19), S-specific IgG in asymptomatic subjects were comparable to that of symptomatic patients sampled within the same time. This is not surprising when considering that subclinical infections have similar or lower infectivity than symptomatic ones (20) and the virus may persist for several weeks after infection (21).

The effect of the antiviral antibody response in COVID-19 is a matter of debate. SARS-CoV-2 infection in rhesus macaques induced immune responses protecting from reinfection (22) and the lack of viral-specific IgG response correlated with poor outcome in severe patients (23). In contrast, we found that S1-IgG levels had a negative effect on the risk of hospitalization. Beyond the breadth of the antibody response, the association between nAbs and survival is controversial. High titers of nAbs were associated with poor outcome (17, 24, 25), whereas the early development of nAbs within the first weeks PSO was critical for patient survival and virus control (26). We found that baseline antibody levels (except S2-IgM) addressed the neutralizing response in COVID-19 patients, but we did not identify any effect on survival (data not shown).

IgA levels dictated the early SARS-CoV-2 antibody response in our cohort. Virus-specific IgA developed within 30 days in moderate and severe patients, together with S1-specific IgM and IgG and NP-reactive IgG. One remarkable finding was that both IgM and IgA targeting S1 strongly correlated with neutralization within 7 days PSO, with the strongest contribution of IgA maintained up to 30 days. The neutralizing potential of IgM and IgA was reported in SARS-CoV-2 infection (27–29). Although neutralizing S-specific IgG are known, the neutralizing potency of monomeric IgA was higher than that of monomeric IgG (30). Conversely, monomeric IgA reduced their neutralizing potential compared with IgG counterpart in recovered patients (31). Several factors might contribute to the poor outcome of COVID-19 in our patients, albeit they developed nAbs as soon as symptoms appeared. First, at the beginning of the outbreak, the spectrum of effective therapeutic interventions was limited. Secondly, the exuberant production of inflammatory cytokine in severe conditions might contribute to the over-production of IgA. Antibodies may function as a double-edged sword acting as a protective mechanism to control the infection or as a harmful process exacerbating the disease. Indeed, nonneutralizing or low-affinity nAbs might have detrimental effect through antibody-dependent enhancing, as shown for other respiratory infections (32–35).

IgA targeting S1 emerged as a good surrogate marker to discriminate the broad variety of COVID-19 manifestations early PSO driving the decision for the appropriate management of patients. Different classes of biomarkers have been identified (10). To the best of our knowledge, this is the first study showing that S1-reactive IgA score may improve risk stratification by using statistical models to examine the impact of several variables on specific outcomes. This unconventional data-driven approach allowed identifying novel correlates of protection, in agreement with the consideration that binding antibodies are “good as a correlate-if not better,” recently pointed out by Cohen (36).

The persistence of SARS-CoV-2-specific antibodies is an open question. Our longitudinal study confirmed that S1-reactive IgG dramatically dropped but remained detectable in all patients even after 8 months PSO, as previously reported (37–39). One remarkable finding was that S2-IgG enhanced over the same time. Nguyen-Contant et al. demonstrated markedly increased levels of S2-IgG in unexposed and convalescent individuals with S2-reactive memory B cells, probably related to pre-exposure to human coronaviruses (40). We can speculate that SARS-CoV-2 infection generated IgG memory B cells reactive to S2 that cross-reacted with the S2 of seasonal human coronaviruses, due to the higher homology of S2 than S1 across human coronaviruses (41).

Nowadays, the question has arisen whether the antibody response to SARS-CoV-2 generated during the first wave of the pandemic is effective against the emerging variant of concern (VOC). The B.1.1.7 and B.1.351, first detected in December 2020 in the UK (42) and South Africa (43), respectively, quickly spread worldwide since extensive mutations on the spike enhanced viral transmission. Our and other emerging studies (44, 45) showed that antibodies raised against the D614G lineage cross-neutralized the two VOCs but at reduced potency, that was marked for the B.1.351 variant. These evidences pointed out the urgent need to implement currently available vaccine formulations to protect against newly emerging VOC. Overall, this study provided new evidences to open opportunities for the early management of infected patients as soon as they are diagnosed and for implementing the current vaccination strategies.

## Data Availability Statement

The original contributions presented in the study are included in the article/**Supplementary Material**. Further inquiries can be directed to the corresponding authors.

## Ethics Statement

The studies involving human participants were reviewed and approved by San Raffaele Scientific Hospital Ethical Committee. Written informed consent for participation was not required for this study in accordance with the national legislation and the institutional requirements.

## Author Contributions

GSi and CBro wrote the manuscript and contributed equally to the work. GSi, CP, and DP performed and analyzed laboratory tests. DC performed some neutralization assays. CBro and FC did the statistical analysis. LL conceived and designed the study. NMau selected and provided control samples. CBoz and GSa selected and provided samples from healthcare workers. MN, ET, MM, and CU-F selected and provided samples from COVID-BioB-San Raffaele Hospital. CBon oversaw the study. NT and DC provided plasmids. NC and NMan performed virological analysis. LL and CBon acquired the funding. CS and LL reviewed the manuscript. All authors reviewed the manuscript for intellectual content and approved the submitted manuscript.

## Funding

The work was funded by Ministero Salute-Italy (COVID-2020-12371617), Scientific Direction of San Raffaele Scientific Institute (Immuno-COVID) and by ANR-France (MUCOLUNG).

## Conflict of Interest

The authors declare that the research was conducted in the absence of any commercial or financial relationships that could be construed as a potential conflict of interest.

## Publisher’s Note

All claims expressed in this article are solely those of the authors and do not necessarily represent those of their affiliated organizations, or those of the publisher, the editors and the reviewers. Any product that may be evaluated in this article, or claim that may be made by its manufacturer, is not guaranteed or endorsed by the publisher.

## Supplementary Material

Supplementary Figure 1 Spearman’s correlations between time to symptom resolution from bleeding and SARS-CoV-2-specific antibodies in nonhospitalized (*n* = 36) subjects.
