# Supplementary figures and images for: Profiling Antibody Response Patterns in COVID-19: Spike S1-Reactive IgA Signature in the Evolution of SARS-CoV-2 Infection
Source: Front Immunol. 2021 Nov 3;12:772239. doi: 10.3389/fimmu.2021.772239 (PMC8595940; doi:10.3389/fimmu.2021.772239)

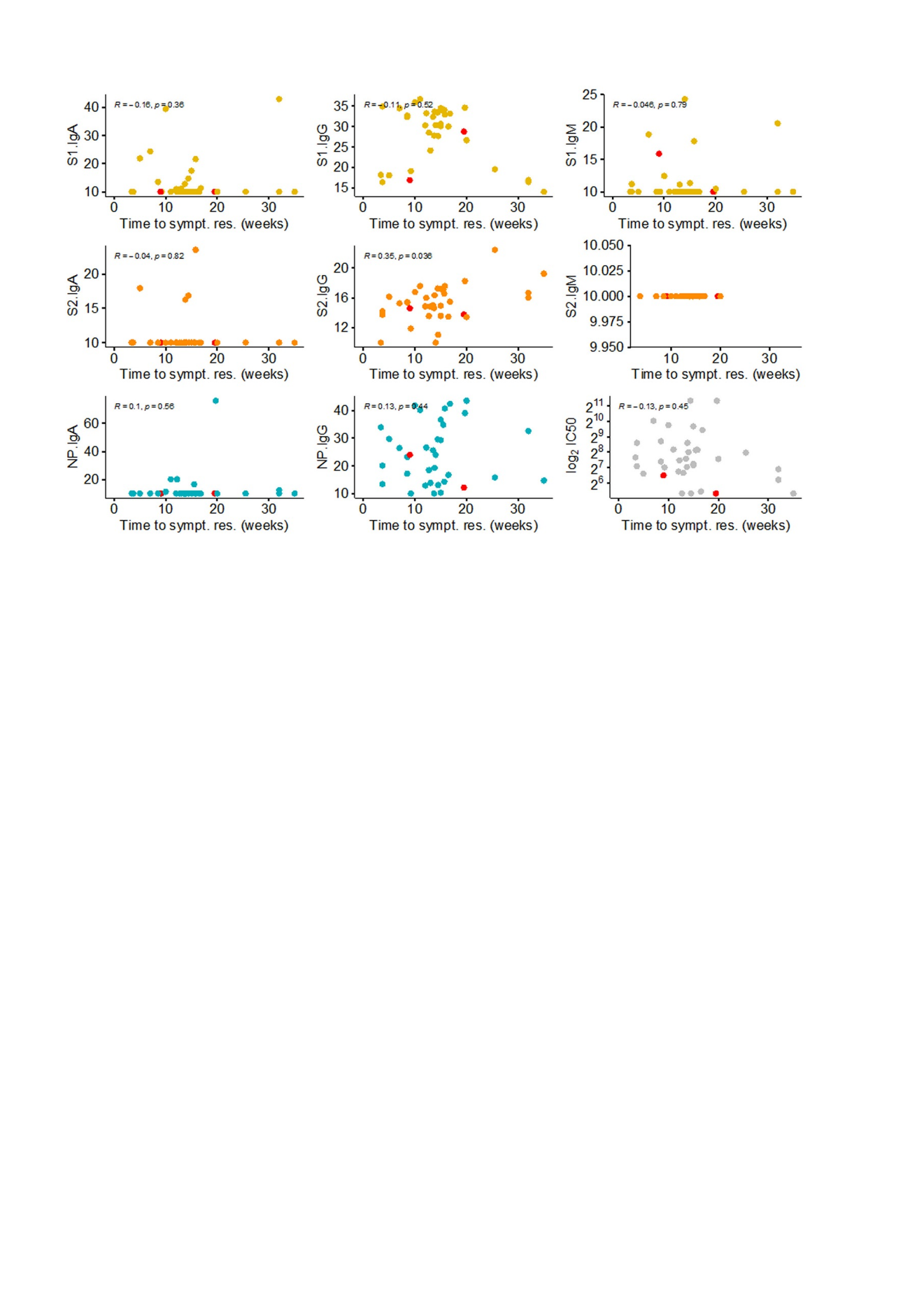

Supplement: Supplementary Figure 1 — Spearman’s correlations between time to symptom resolution from bleeding and SARS-CoV-2-specific antibodies in nonhospitalized (n = 36) subjects. [file Image_1.tif]
