# Supplementary material for: Profiling Antibody Response Patterns in COVID-19: Spike S1-Reactive IgA Signature in the Evolution of SARS-CoV-2 Infection
Source: Front Immunol. 2021 Nov 3;12:772239. doi: 10.3389/fimmu.2021.772239 (PMC8595940; doi:10.3389/fimmu.2021.772239)
Supplement: Supplementary file 2 [file DataSheet_1.pdf]

Patient selection and data collection.
The inclusion criterion for this clinical-biological case series study was confirmed COVID-19 infection, defined as positive SARS-CoV-2 PCR from nasal/throat swab and radiological findings suggestive of COVID-19 pneumonia. Patients were selected according to a six-category ordinal scale of clinical status ranging from no requirement of hospital care to death (64). The use of a categorical endpoint for clinical outcome evaluation has been recommended by WHO R&D Blueprint expert group to facilitate interpretation and combination of results across studies and trials (2). A categorical descriptor was applied based on this scoring system: "mild" for a score of 1-2, "moderate" for a score of 3-4, and "severe" for a score of 5-6.
Production and titration of SARS-CoV-2 lentiviral pseudotyped particles.
Briefly, HEK293 T/17 (at the 60% of confluence) were co-transfected with the S plasmids from SARS-CoV-2, HIV gag-pol and the pCSFLW using FuGENE® HD Transfection Reagent (Promega) in accordance with the manufacturer's instructions. Pseudotype containing supernatant was harvested 72h post−transfection, centrifuged at 500xg for 5 min to clear cell debris and filtered with a 0.45-mm filter. Aliquots were stored at -80°C. For titration of pseudotypes stock and the neutralization assay, HEK 293T/17 cells were transfected with pACE2 and pTMPRSS2 for 24 h. Virus infectivity was determined by titration on HEK 293T/17-ACE2/TMPRSS2 cells as previously described. Plasma samples were heat inactivated by incubation at 56°C for 30 min.
Statistical Analysis.
Descriptive statistics for SARS-CoV-2 antibodies and IC50 have been expressed as median and interquartile range, minimum and maximum, as well as frequency and percentage showing values below/above the threshold of "positivity". Spearman's correlation coefficient has been computed to examine the relationship among quantitative variables, whenever sample size was large enough for carrying our sensible inferential procedures.
In presence of repeated measures, Friedman test followed by Dunn's multiple comparison tests has been applied.
Tobit regression models (3) have been estimated to (i) evaluate the impact of both clinical and demographic characteristics on SARS-CoV-2 antibodies and to (ii) explore the relationship between antibody response (considered as a binary variable, hence positive/negative) on IC50, while accounting for clinical and demographic covariates. This modelling approach is suited for modelling left censored dependent variable. Logarithmic transformation of IC50 (base 2) was considered to reduce the excess of skewness/kurtosis in the collected distribution.
Classification and Regression Trees (CART) analyses have been performed to identify those variables that best discriminate patients with different outcomes (either hospitalization or disease severity). CART is a nonparametric data-driven approach which allows to deal with mixed types of variables (continuous and/or categorical) while requiring less stringent assumptions than standard modelling procedures. CART implements a binary recursive partitioning where parent nodes are always split into two child nodes and the process is recursively repeated by treating each child node as a parent (4). The procedure automatically selects variables and the corresponding cut-off values that best differentiate observations with different outcome variable. The underlying criterion used in classification trees is the GINI rule.
To examine the dynamic of IC50 over time and in patients grouped according to their positive or negative antibody response at baseline, while accounting for age and gender, Linear Mixed-Effects (LME, 5) models have been applied. In particular, grouping variable (indicating whether a patient has positive/negative antibody response at baseline) and time variable (a categorical variable taking three values: T0 if the time of sampling from symptoms onset was lower than 30 days, T1 if the time was comprised between 30 and 60 days, T2 if the time was larger than 60 days) were entered in the model as main effects as well as in interaction. This modelling approach properly accounts for the dependency among observations arising from measuring the same subjects more than once and over time. To account for patients-specific heterogeneity, a random effect was specified on patients ID, thus leading to estimate random intercept models. Logarithmic transformation of IC50 was also considered to satisfy underlying model assumptions. Backward model selection procedures have been applied to obtain a smaller set of relevant covariates. After model selection, post-hoc analyses have been implemented to compare IC50 response in the two groups of patients (with positive or negative antibody response at baseline respectively) at a fixed time point.
All the analyses were performed using R statistical software (version 4.0.4). The significance level was set at 0.05. The R packages rpart and VGAM have been used to implement respectively the classification tree analysis and the Tobit model. LME were estimated using the nlme package and post-hoc comparisons have been performed using emmeans package.

References

1. Y. Wang, *et al.*, Remdesivir in adults with severe COVID-19: a randomised, double-blind, placebo-controlled, multicentre trial. *Lancet* **395**, 1569–1578 (2020).
2. COVID, WHO (2020) Therapeutic Trial Synopsis. https://www.who.int/publications/i/item/covid-19-therapeutic-trial-synopsis
3. J. Tobin. Estimation of relationships for limited dependent variables. Econometrica 26: 24-36. (1958).
4. L. Breiman, *et al.*, Classification and regression trees. CRC press. (1984).
5. J. C. Pinheiro, D. M. Bates. Linear mixed-effects models: basic concepts and examples. *Mixed-effects models in S and S-Plus*, 3-56. (2000).
