# Supplementary material for: Profiling Antibody Response Patterns in COVID-19: Spike S1-Reactive IgA Signature in the Evolution of SARS-CoV-2 Infection
Source: Front Immunol. 2021 Nov 3;12:772239. doi: 10.3389/fimmu.2021.772239 (PMC8595940; doi:10.3389/fimmu.2021.772239)
Supplement: Supplementary file 3 [file Table_1.docx]

**Table S1**. SARS-CoV-2 antibodies levels and neutralizing antibodies titers in asymptomatic (n=19) and symptomatic subjects (n=122).

|  |  | **asymptomatic**  **(n=19)** | **0-7 days**  **(n=23)** | **8-30 days**  **(n=31)** | **31-60 days**  **(n=21)** | **2-4 months**  **(n=28)** | **5-9 months**  **(n=19)** |
| --- | --- | --- | --- | --- | --- | --- | --- |
|  |  |  |  |  |  |  |  |
| **Anti-S1 IgA** | Median [IQR] | 11.48  [10, 16] | 12.68  [10, 36.16] | 15.67  [10, 28.84] | 19.28  [10, 31.93] | 10  [10, 15.91] | 10  [10, 15.09] |
|  | ≤10 - n.(%) | 7 ( 36.8) | 10 (43.5) | 14 (45.2) | 6 (28.6) | 15 (53.6) | 11 ( 57.9) |
|  | >10 - n.(%) | 12 ( 63.2) | 13 (56.5) | 17 (54.8) | 15 (71.4) | 13 (46.4) | 8 ( 42.1) |
| **Anti-S2 IgA** | Median [IQR] | 10  [10, 10] | 10  [10, 12.00] | 10  [10, 20.71] | 10  [10, 16.73] | 10  [10, 10] | 10  [10, 10] |
|  | ≤10 - n.(%) | 17 ( 89.5) | 15 (65.2) | 19 (61.3) | 11 (52.4) | 25 (89.3) | 17 ( 89.5) |
|  | >10 - n.(%) | 2 ( 10.5) | 8 (34.8) | 12 (38.7) | 10 (47.6) | 3 (10.7) | 2 ( 10.5) |
| **Anti-NP IgA** | Median [IQR] | 10  [10, 10] | 14.78  [10, 125.91] | 32.63  [11.66, 52.94] | 25.63  [10, 40.40] | 10  [10, 10.51] | 10  [10, 11.80] |
|  | ≤10 - n.(%) | 17 ( 89.5) | 10 (43.5) | 8 (25.8) | 10 (47.6) | 20 (71.4) | 13 ( 68.4) |
|  | >10 - n.(%) | 2 ( 10.5) | 13 (56.5) | 23 (74.2) | 11 (52.4) | 8 (28.6) | 6 ( 31.6) |
| **Anti S1 IgG** | Median [IQR] | 18.11  [11.02, 23.94] | 10  [10, 18.40] | 12.89  [10, 17.11] | 18.38  [16.7, 29.66] | 31.49  [27.25, 33.91] | 26.65  [19.32, 33.53] |
|  | ≤10 - n.(%) | 5 ( 26.3) | 12 (52.2) | 14 (45.2) | 1 ( 4.8) | 2 ( 7.1) | 0 |
|  | >10 - n.(%) | 14 ( 73.7) | 11 (47.8) | 17 (54.8) | 20 (95.2) | 26 (92.9) | 19 (100.0) |
| **Anti S2 IgG** | Median [IQR] | 12.51  [11.01, 16.86] | 12.57  [10, 15.14] | 13.68  [10, 16.25] | 15.62  [14.19, 16.85] | 15.32  [14.22, 16.84] | 16.64  [15.16, 18.20] |
|  | ≤10 - n.(%) | 4 ( 21.1) | 9 (39.1) | 10 (32.3) | 2 ( 9.5) | 3 (10.7) | 0 |
|  | >10 - n.(%) | 15 ( 78.9) | 14 (60.9) | 21 (67.7) | 19 (90.5) | 25 (89.3) | 19 (100.0) |
| **Anti NP IgG** | Median [IQR] | 10  [10, 21.46] | 21.98  [10, 27.49] | 20.99  [10, 27.53] | 24.23  [19.52, 29.68] | 23.51  [13.94, 29.38] | 29.55  [15.23, 39.58] |
|  | ≤10 - n.(%) | 10 ( 52.6) | 9 (39.1) | 10 (32.3) | 2 ( 9.5) | 4 (14.3) | 2 ( 10.5) |
|  | >10 - n.(%) | 9 ( 47.4) | 14 (60.9) | 21 (67.7) | 19 (90.5) | 24 (85.7) | 17 ( 89.5) |
| **Anti S1 IgM** | Median [IQR] | 10  [10, 10] | 12.17  [10, 19.77] | 12.34  [10, 23.49] | 10  [10, 14.91] | 10  [10, 15.69] | 10  [10, 10] |
|  | ≤10 - n.(%) | 16 ( 84.2) | 11 (47.8) | 11 (35.5) | 12 (57.1) | 18 (64.3) | 15 ( 78.9) |
|  | >10 - n.(%) | 3 ( 15.8) | 12 (52.2) | 20 (64.5) | 9 (42.9) | 10 (35.7) | 4 ( 21.1) |
| **Anti S2 IgM** | Median [IQR] | 10  [10, 10] | 10  [10, 11.76] | 10  [10, 10] | 10  [10, 10] | 10  [10, 10] | 10  [10, 10] |
|  | ≤10 - n.(%) | 8 (100.0) | 15 (68.2) | 23 (76.7) | 15 (83.3) | 27 (96.4) | 11 ( 91.7) |
|  | >10 - n.(%) | 0 | 7 (31.8) | 7 (23.3) | 3 (16.7) | 1 ( 3.6) | 1 ( 8.3) |
| **Anti NP IgM** | Median [IQR] | 10  [10, 10] | 10  [10, 13.15] | 11.63  [10, 17.99] | 10  [10, 10] | 10  [10, 11.71] | - |
|  | ≤10 - n.(%) | 3 (100.0) | 9 (69.2) | 7 (50.0) | 13 (92.9) | 2 (66.7) | - |
|  | >10 - n.(%) | 0 ( 0.0) | 4 (30.8) | 7 (50.0) | 1 ( 7.1) | 1 (33.3) | - |
| **IC50** | Median [IQR] | 254  [42, 476.53] | 154  [40, 647.58] | 557  [147.1, 1628.5] | 483  [207.29, 1957] | 223.01  [125.16, 681.94] | 242  [87.47, 459.15] |
|  | ≤40 - n.(%) | 5 (26.3) | 8 (34.8) | 5 (16.1) | 1 (4.8) | 4 (14.3) | 4 (21.1) |
|  | >40 - n.(%) | 14 (73.7) | 15 (65.2) | 26 (83.9) | 20 (95.2) | 24 (85.7) | 15 (78.9) |
