# Supplementary material for: Profiling Antibody Response Patterns in COVID-19: Spike S1-Reactive IgA Signature in the Evolution of SARS-CoV-2 Infection
Source: Front Immunol. 2021 Nov 3;12:772239. doi: 10.3389/fimmu.2021.772239 (PMC8595940; doi:10.3389/fimmu.2021.772239)
Supplement: Supplementary file 4 [file Table_2.docx]

**Table S2: Tobit models for investigating the role of undetectable or detectable antibody levels (values below the cut-off, ≤10; values above the cut-off, >10, respectively) on neutralization activity as measured by IC50**.

|  | **Estimate** | **Std. Error** | **p-value** |
| --- | --- | --- | --- |
| Intercept:1 | 3.1929 | 0.6922 | **<0.0001** |
| Intercept:2 | 0.4256 | 0.0712 | **<0.0001** |
| Anti-S1 IgA (>10 vs. ≤10) | 1.9118 | 0.3074 | **<0.0001** |
| Hospitalization (yes vs. no) | -0.0764 | 0.4268 | 0.8579 |
| Time from disease onset (days) | -0.0011 | 0.003 | 0.7126 |
| Age | 0.0224 | 0.0116 | 0.0537 |
| Sex (Male vs. Female) | 0.4484 | 0.2975 | 0.1318 |

|  | **Estimate** | **Std. Error** | **p-value** |
| --- | --- | --- | --- |
| Intercept:1 | 3.1915 | 0.7183 | **<0.0001** |
| Intercept:2 | 0.4636 | 0.0712 | **<0.0001** |
| Anti-S2 IgA (>10 vs. ≤10) | 1.7268 | 0.3519 | **<0.0001** |
| Hospitalization (yes vs. no) | 0.0322 | 0.4435 | 0.9421 |
| Time from disease onset (days) | 0.0015 | 0.0031 | 0.6427 |
| Age | 0.0292 | 0.0119 | **0.014** |
| Sex (Male vs. Female) | 0.3258 | 0.3087 | 0.2912 |

|  | **Estimate** | **Std. Error** | **p-value** |
| --- | --- | --- | --- |
| Intercept:1 | 3.4551 | 0.7585 | **<0.0001** |
| Intercept:2 | 0.5027 | 0.0714 | **<0.0001** |
| Anti-NP IgA (>10 vs. ≤10) | 1.5341 | 0.3863 | **0.0001** |
| Hospitalization (yes vs. no) | -0.3658 | 0.4951 | 0.46 |
| Time from disease onset (days) | -9e-04 | 0.0033 | 0.7956 |
| Age | 0.0268 | 0.0126 | **0.033** |
| Sex (Male vs. Female) | 0.2581 | 0.3232 | 0.4246 |

|  | **Estimate** | **Std. Error** | **p-value** |
| --- | --- | --- | --- |
| Intercept:1 | 1.523 | 0.6794 | **0.025** |
| Intercept:2 | 0.3544 | 0.0709 | **<0.0001** |
| Anti-S1 IgG (>10 vs. ≤10) | 2.743 | 0.357 | **<0.0001** |
| Hospitalization (yes vs. no) | 0.9296 | 0.3989 | **0.0198** |
| Time from disease onset (days) | -0.0055 | 0.0029 | 0.061 |
| Age | 0.028 | 0.0106 | **0.0084** |
| Sex (Male vs. Female) | 0.3603 | 0.2771 | 0.1935 |

|  | **Estimate** | **Std. Error** | **p-value** |
| --- | --- | --- | --- |
| Intercept:1 | 2.2402 | 0.7719 | **0.0037** |
| Intercept:2 | 0.5 | 0.0712 | **<0.0001** |
| Anti-S2 IgG (>10 vs. ≤10) | 1.6227 | 0.4176 | **0.0001** |
| Hospitalization (yes vs. no) | 0.4527 | 0.4536 | 0.3183 |
| Time from disease onset (days) | -0.0032 | 0.0034 | 0.3475 |
| Age | 0.0315 | 0.0123 | **0.0104** |
| Sex (Male vs. Female) | 0.3872 | 0.3201 | 0.2264 |

|  | **Estimate** | **Std. Error** | **p-value** |
| --- | --- | --- | --- |
| Intercept:1 | 2.5687 | 0.7203 | **0.0004** |
| Intercept:2 | 0.4413 | 0.0708 | **<0.0001** |
| Anti-NP IgG (>10 vs. ≤10) | 2.2062 | 0.3903 | **<0.0001** |
| Hospitalization (yes vs. no) | 0.6062 | 0.43 | 0.1586 |
| Time from disease onset (days) | -0.0037 | 0.0032 | 0.2488 |
| Age | 0.0192 | 0.012 | 0.1087 |
| Sex (Male vs. Female) | 0.196 | 0.3039 | 0.5189 |

|  | **Estimate** | **Std. Error** | **p-value** |
| --- | --- | --- | --- |
| Intercept:1 | 2.9355 | 0.7456 | **0.0001** |
| Intercept:2 | 0.5008 | 0.0713 | **<0.0001** |
| Anti-S1 IgM (>10 vs. ≤10) | 1.2963 | 0.3322 | **0.0001** |
| Hospitalization (yes vs. no) | 0.4876 | 0.4531 | 0.2819 |
| Time from disease onset (days) | 0.0028 | 0.0033 | 0.4041 |
| Age | 0.0273 | 0.0125 | **0.0288** |
| Sex (Male vs. Female) | 0.1662 | 0.3264 | 0.6106 |

|  | **Estimate** | **Std. Error** | **p-value** |
| --- | --- | --- | --- |
| Intercept:1 | 1.7901 | 0.9783 | 0.0673 |
| Intercept:2 | 0.5839 | 0.075 | **<0.0001** |
| Anti-S2 IgM (>10 vs. ≤10) | 0.2971 | 0.4827 | 0.5382 |
| Hospitalization (yes vs. no) | 0.7254 | 0.5563 | 0.1923 |
| Time from disease onset (days) | 0.0099 | 0.0054 | 0.0682 |
| Age | 0.045 | 0.0143 | **0.0016** |
| Sex (Male vs. Female) | 0.5573 | 0.3745 | 0.1367 |

|  | **Estimate** | **Std. Error** | **p-value** |
| --- | --- | --- | --- |
| Intercept:1 | 1.4339 | 1.4794 | 0.3324 |
| Intercept:2 | 0.6223 | 0.1159 | **<0.0001** |
| Anti-NP IgM (>10 vs. ≤10) | 1.4028 | 0.6676 | **0.0356** |
| Hospitalization (yes vs. no) | 0.6317 | 1.0488 | 0.5469 |
| Time from disease onset (days) | 0.0248 | 0.015 | 0.0966 |
| Age | 0.0469 | 0.022 | **0.0328** |
| Sex (Male vs. Female) | 0.2203 | 0.6065 | 0.7164 |
