# Supplementary material for: Profiling Antibody Response Patterns in COVID-19: Spike S1-Reactive IgA Signature in the Evolution of SARS-CoV-2 Infection
Source: Front Immunol. 2021 Nov 3;12:772239. doi: 10.3389/fimmu.2021.772239 (PMC8595940; doi:10.3389/fimmu.2021.772239)
Supplement: Supplementary file 5 [file Table_3.docx]

**Table S3: Tobit models for investigating the role of hospitalization in predicting specific SARS-CoV-2 antibody responses and neutralization activity while accounting for age, sex, time from disease onset**

| **Anti-S1.IgA** | **Estimate** | **Std. Error** | **p-value** |
| --- | --- | --- | --- |
| Intercept:1 | -24.6138 | 9.3409 | **0.0084** |
| Intercept:2 | 2.8865 | 0.0849 | **<0.0001** |
| Hospitalization (yes vs. no) | 16.1111 | 5.5137 | 0.0035 |
| Time from disease onset (days) | 0.0494 | 0.0391 | 0.207 |
| Age | 0.3903 | 0.1446 | 0.007 |
| Sex (Male vs. Female) | 2.9153 | 3.7773 | 0.4402 |

| **Anti-S2.IgA** | **Estimate** | **Std. Error** | **p-value** |
| --- | --- | --- | --- |
| Intercept:1 | -27.3801 | 11.3207 | **0.0156** |
| Intercept:2 | 2.8587 | 0.0957 | **<0.0001** |
| Hospitalization (yes vs. no) | 12.5185 | 6.7448 | 0.0634 |
| Time from disease onset (days) | -0.0383 | 0.0509 | 0.4518 |
| Age | 0.3208 | 0.1666 | 0.0542 |
| Sex (Male vs. Female) | 3.0541 | 4.3801 | 0.4856 |

| **Anti-NP.IgA** | **Estimate** | **Std. Error** | **p-value** |
| --- | --- | --- | --- |
| Intercept:1 | -1875.0472 | 461.1359 | **<0.0001** |
| Intercept:2 | 6.6119 | 0.092 | **<0.0001** |
| Hospitalization (yes vs. no) | 833.0745 | 274.2956 | **0.0024** |
| Time from disease onset (days) | 0.977 | 1.8945 | 0.6061 |
| Age | 17.6125 | 6.7777 | **0.0094** |
| Sex (Male vs. Female) | -89.5946 | 176.7707 | 0.6123 |

| **Anti-S1.IgG** | **Estimate** | **Std. Error** | **p-value** |
| --- | --- | --- | --- |
| Intercept:1 | 17.7797 | 4.3362 | **<0.0001** |
| Intercept:2 | 2.2688 | 0.0738 | **<0.0001** |
| Hospitalization (yes vs. no) | -7.3695 | 2.643 | **0.0053** |
| Time from disease onset (days) | 0.0548 | 0.0188 | **0.0036** |
| Age | 0.0452 | 0.0721 | 0.5309 |
| Sex (Male vs. Female) | 0.3616 | 1.8918 | 0.8484 |

| **Anti-S2.IgG** | **Estimate** | **Std. Error** | **p-value** |
| --- | --- | --- | --- |
| Intercept:1 | 8.6698 | 1.6239 | **<0.0001** |
| Intercept:2 | 1.2858 | 0.0708 | **<0.0001** |
| Hospitalization (yes vs. no) | 1.3896 | 0.9883 | 0.1597 |
| Time from disease onset (days) | 0.0337 | 0.0071 | **<0.0001** |
| Age | 0.0455 | 0.0266 | 0.0868 |
| Sex (Male vs. Female) | 0.3594 | 0.6992 | 0.6072 |

| **Anti-NP.IgG** | **Estimate** | **Std. Error** | **p-value** |
| --- | --- | --- | --- |
| Intercept:1 | -2.3257 | 6.2421 | 0.7095 |
| Intercept:2 | 2.611 | 0.0746 | **<0.0001** |
| Hospitalization (yes vs. no) | 3.028 | 3.7669 | 0.4215 |
| Time from disease onset (days) | 0.0782 | 0.0269 | **0.0037** |
| Age | 0.2664 | 0.1015 | **0.0087** |
| Sex (Male vs. Female) | 4.1182 | 2.6603 | 0.1216 |

| **Anti-S1.IgM** | **Estimate** | **Std. Error** | **p-value** |
| --- | --- | --- | --- |
| Intercept:1 | -7.3335 | 6.6723 | 0.2717 |
| Intercept:2 | 2.509 | 0.0899 | **<0.0001** |
| Hospitalization (yes vs. no) | -0.468 | 4.0314 | 0.9076 |
| Time from disease onset (days) | -0.0662 | 0.032 | **0.0385** |
| Age | 0.2762 | 0.1057 | **0.009** |
| Sex (Male vs. Female) | 5.9445 | 2.7342 | **0.0297** |

| **Anti-S2.IgM** | **Estimate** | **Std. Error** | **p-value** |
| --- | --- | --- | --- |
| Intercept:1 | 3.5571 | 5.4541 | 0.5143 |
| Intercept:2 | 1.9203 | 0.1085 | **<0.0001** |
| Hospitalization (yes vs. no) | 4.8874 | 3.7625 | 0.1939 |
| Time from disease onset (days) | -0.0644 | 0.0357 | 0.0716 |
| Age | -0.0012 | 0.0799 | 0.9881 |
| Sex (Male vs. Female) | -2.3969 | 2.1715 | 0.2697 |

| **Anti-NP.IgM** | **Estimate** | **Std. Error** | **p-value** |
| --- | --- | --- | --- |
| Intercept:1 | -10.5802 | 13.8676 | 0.4455 |
| Intercept:2 | 2.5528 | 0.1569 | **<0.0001** |
| Hospitalization (yes vs. no) | -4.1969 | 9.4681 | 0.6576 |
| Time from disease onset (days) | -0.3357 | 0.1398 | **0.0163** |
| Age | 0.2875 | 0.1893 | 0.129 |
| Sex (Male vs. Female) | 10.2966 | 5.4602 | 0.0593 |

| **log_2_IC50** | **Estimate** | **Std. Error** | **p-value** |
| --- | --- | --- | --- |
| Intercept:1 | 4.1531 | 1.147 | **0.0003** |
| Intercept:2 | 0.9322 | 0.0716 | **<0.0001** |
| Hospitalization (yes vs. no) | 0.6 | 0.696 | 0.3886 |
| Time from disease onset (days) | 9e-04 | 0.005 | 0.8544 |
| Age | 0.0561 | 0.0188 | **0.0028** |
| Sex (Male vs. Female) | 0.6234 | 0.4917 | 0.2049 |
